# Predictive validity of the N2 and P3 ERP components to executive functioning in children: a latent-variable analysis

**DOI:** 10.3389/fnhum.2014.00080

**Published:** 2014-02-20

**Authors:** Christopher R. Brydges, Allison M. Fox, Corinne L. Reid, Mike Anderson

**Affiliations:** ^1^Neurocognitive Development Unit, School of Psychology, The University of Western AustraliaPerth, WA, Australia; ^2^School of Psychology and Exercise Science, Murdoch UniversityPerth, WA, Australia

**Keywords:** executive functions, children, working memory, inhibition, cognitive control, ERP, N2, P3

## Abstract

Executive functions (EFs) are commonly theorized to be related yet separable constructs in adults, and specific EFs, such as prepotent response inhibition and working memory, are thought to have clear and distinct neural underpinnings. However, recent evidence suggests that EFs are unitary in children up to about 9 years of age. The aim of the current study was to test the hypothesis that peaks of the event-related potential (ERP) of specific EFs are related to behavioral performance, despite EFs being psychometrically indistinguishable in children. Specifically, N2 difference waveform (associated with cognitive control and response inhibition) and P3b peak (associated with updating of working memory) latent variables were created and entered into confirmatory factor analysis and structural equation models with a unitary executive functioning factor. Children aged 7–9 years (*N* = 215) completed eight measures of inhibition, working memory, and shifting. A modified flanker task was also completed during which EEG data were recorded. The N2 difference waveform and P3b mean amplitude factors both significantly correlated with (and were predictors of) the executive functioning factor, but the P3b latency factor did not. These results provide evidence of the electrophysiological indices of EFs being observable before the associated behavioral constructs are distinguishable from each other. From this, it is possible that ERPs could be used as a sensitive measure of development in the context of evaluation for neuropsychological interventions.

## Introduction

Executive functions are higher-order cognitive functions that are associated with goal-directed behavior (Miller and Cohen, 2001). The development of executive functions throughout childhood is of critical importance, as these functions are associated with academic achievement in children (St Clair-Thompson and Gathercole, 2006) and successful living (Garavan et al., 1999). Previous research has found electrophysiological correlates of specific executive functions in both adults and children (Polich et al., 1990; Van Veen and Carter, 2002; Walhovd and Fjell, 2002; Cragg et al., 2009; Krug and Carter, 2012), providing evidence of distinct neural substrates of these processes. However, these studies have not taken differences in the structure of executive functions between adults and children into account (Miyake et al., 2000; Lehto et al., 2003; Brydges et al., 2012b). Executive functions in adults are generally considered to be related yet separable constructs (Miyake et al., 2000); however, recent psychometric evidence suggests that the latent traits of inhibition, working memory, and shifting are indistinguishable from each other in typically developing children up to at least the age of 9 years (Brydges et al., 2012b). Given this, the current study aims to determine how these distinct components in the event-related potential (ERP) are related to aspects of executive functioning when incompletely developed in children.

One widely accepted model of executive functions was initially proposed by Miyake et al. (2000), who used confirmatory factor analysis (CFA) on multiple measures of three commonly postulated executive functions (prepotent response inhibition, updating of working memory, and task switching). The use of CFA in this context is advantageous because measures of executive functions all have some degree of task impurity (Rabbitt, 1997). Non-executive processes (such as motor control) are a necessary part of any task that is designed to measure executive functioning. CFA alleviates this problem by using several measures of each executive function and extracting the common variance between these measures, to create a “pure” latent variable, or factor, which can then be correlated with other factors. The resultant model reported by Miyake et al. provided evidence these three constructs were found to be related yet are distinct from one another, as evidenced by moderately strong correlations between each factor, ranging from *r* = 0.42 to *r* = 0.63.

Several studies have attempted to replicate the Miyake et al. (2000) model of executive functions in children. In young children, these executive functions are indistinguishable, resulting in a unitary model of executive functioning in children at least up to the age of 9 years (Wiebe et al., 2008; Hughes et al., 2009; Brydges et al., 2012b; Willoughby et al., 2012). Hughes et al. conducted a longitudinal study to examine the development of executive functions in young children, and reported a single factor was the best fit for the data at both 4 and 6 years of age. Additionally, Brydges et al. tested a group of 7-year-old and a group of 9-year-old children, and found that the structure of executive functions was invariant between groups, despite improved performance with age. However, as children develop past the age of 9 years, these executive functions are thought to become increasingly separable. Lehto et al. (2003) reported three related yet separable executive functions in children aged 8–13 years (mean age of 10.5 years). Furthermore, Wu et al. (2011) and Duan et al. (2010) also both reported unity and diversity of executive functions in older children (mean ages of 10.9 years and 11.8 years respectively). Hence, it is possible that executive functions develop globally until about 9 years of age, before differentiation occurs in mid to late childhood. It should be noted, however, that the age at which executive functions are distinguishable is subject to some variation, possibly due to the nature of the tasks used in each study (Van Der Sluis et al., 2007).

To further knowledge regarding the links between brain and behavior, previous research in both adults and children has attempted to examine the relationship between specific neural processes associated with executive functions and behavioral performance on psychometric measures of these functions (Rushworth et al., 2002; Van Veen and Carter, 2002; Polich, 2007; Krug and Carter, 2012). Two components of direct relevance to the model of executive functions described above are the N2 and P3b peaks of the ERP.

The N2 peak is a fronto-central maximal negativity observed approximately 150–400 ms after stimulus onset (although often later in children), and has been repeatedly associated with the detection of response conflict in both children and adults (Jodo and Kayama, 1992; Van Veen and Carter, 2002; Cragg et al., 2009). Jodo and Kayama used an electroencephalogram to record electrophysiological activity in young adults during a Go/Nogo task, and reported larger N2 amplitudes were associated with fewer errors on Nogo trials. Cragg et al. reported a significantly larger N2 amplitude on Nogo trials than on go trials in typically developing children aged 7–9 years, providing further evidence of the N2 being an electrophysiological correlate of response conflict and inhibition.

The P3b peak is a positivity seen at central and parietal scalp sites approximately 300–500 ms after stimulus onset (again, often observed later in children), and has been associated with updating of working memory (Donchin and Coles, 1988; Polich, 2007). Walhovd and Fjell (2002) found positive associations between P3b amplitude, latency (both obtained during an auditory oddball task at central midline scalp sites) and performance on a digit span task in a sample of adults aged 21–94 years. These relationships were also observed in a sample of children and young adults aged 4–20 years (Polich et al., 1990), further highlighting a link between the P3b and working memory.

The central issue of the current study is that the ERP correlates of executive functions are observable in mid- to late-childhood (Polich et al., 1990; Cragg et al., 2009); however, psychometric research suggests that the latent traits of these functions are not distinguishable from each other during this developmental period (Hughes et al., 2009; Brydges et al., 2012b). From this, it is possible that ERP components develop before specific executive abilities. If associations between ERP components and executive functioning exist, ERPs could potentially be used as a more sensitive measure of neuropsychological development than traditional psychometric measures.

To the authors' knowledge, no previous study has attempted to examine associations between ERPs and executive functions using structural equation modeling (SEM). The current study aimed to determine (a) if there is a correlational association between brain and behavioral measures of executive functions; and (b) if the electrophysiological activity predicts behavioral performance. Hence, it was predicted that both ERP latent variables would significantly correlate with an executive function latent variable in a CFA, and both be significant predictors of the executive function latent variable in a structural equation model.

## Materials and methods

The data used in the current study merges two previously published datasets. The behavioral data have previously been reported in Brydges et al. (2012b), where full descriptions of the participants, procedures, and eight executive functioning measures are provided. ERP data from the Flanker task (described below) of a subset of approximately 120 of these children have also been previously reported by Richardson et al., (unpublished manuscript, The University of Western Australia). Approval for the study was provided by the Human Research Ethics Office of The University of Western Australia. Parents/guardians of the child participants provided written informed consent.

### Participants

Participants were 215 typically developing children aged 7 years 1 month to 9 years 11 months (110 males and 105 females, *M* = 8 years 4 months, *SD* = 1 year 1 month). These children were recruited through Project K.I.D.S. (Kids' Intellectual Development Study) at the Neurocognitive Development Unit of the School of Psychology of the University of Western Australia. Advertisements were placed in newsletters of local schools, and interested parents/guardians were sent screening questionnaires to ensure the eligibility of their child. The measures used were part of a larger battery of tests designed to measure the cognitive, social, and emotional development of the children (Reid and Anderson, 2012). All participants were healthy at the time of testing, reported normal or corrected-to-normal vision and hearing, and had no reported history of neurological or psychiatric conditions. Their WISC-IV (Wechsler, 2003) IQ scores were within normal range (*M* = 107.05, *SD* = 12.63).

### Apparatus

The executive function latent variable was created using performance on the Stroop task (Stroop, 1935), Compatibility Reaction Time, WISC-IV Letter-Number Sequencing (Wechsler, 2003), WISC-IV Backwards Digit Span (Wechsler, 2003), NEPSY Sentence Repetition (Korkman et al., 1997), Wisconsin Card Sorting Test (WCST; Heaton et al., 1993), BAS-II Verbal Fluency (Elliott et al., 1997), and a Letter Monitoring task (Duncan et al., 1996). These tasks were selected as they are commonly regarded as indicators of one of the three executive functions tested in the original Miyake et al. (2000) model. A Go/Nogo task (Cragg et al., 2009) was also administered, but was found to not significantly load onto the executive function factor. Removing the task did not have any effect, so it was excluded from all analyses (descriptive statistics and correlations for this task have been provided for reference in Tables 1 and 2, respectively).

**Table 1:** **Descriptive statistics of executive function and ERP measures before transformation (*N* = 215)**.

**Task**	***M***	***SD***	**Range**
**EXECUTIVE FUNCTIONING**			
Stroop^a^	25.72	14.55	0.00–97.89
Go/no-go^b^	0.45	0.22	0.00–1.00
Compatibility reaction time^c^^*^	155.99	249.37	−811.97–1426.94
Letter-number sequencing^d^	15.18	4.29	4–22
Backward digit span^d^	6.21	1.54	2–11
Sentence repetition^d^	21.67	4.03	2–32
Wisconsin card sorting test^e^	25.87	19.14	4–94
Verbal fluency^f^	21.50	5.44	9–38
Letter monitoring^g^	3.34	1.98	0–6
**ERPs**			
P3b ERP mean amplitude composite (at site Pz)^h^	12.86	7.64	−4.53–35.72
P3b ERP latency composite^i^	1205.51	13.79	1168–1272
N2 difference waveform mean amplitude composite (at site Cz)^h^	−3.46	3.61	−14.39–4.76
N2 difference waveform latency (incongruous—congruous)^i^	387.55	26.08	352–448
N2 difference waveform latency (reversed—congruous)^i^	372.22	31.03	308–460
**FLANKER TASK**			
Congruous condition reaction time^i^	869.05	233.08	450.30–2062.30
Congruous condition accuracy^b^	0.89	0.08	0.59–1.00
Incongruous condition reaction time^i^	1011.40	330.70	481.60–3133.60
Incongruous condition accuracy^b^	0.84	0.13	0.33–1.00
Reversed condition reaction time^i^	1020.07	285.18	569.80–2487.45
Reversed condition accuracy^b^	0.81	0.12	0.26–1.00

a Difference between incongruous and neutral conditions (s).

b Proportion correct.

c Difference between block 5 and blocks 1–4 (ms).

d Total points scored.

e Perseverative errors.

f Number of words.

g Total items correct.

h μV.

i ms.

* Note that the SD for Compatibility Reaction Time are quite high, but decreased after trimming and transformation to −154.79 ms (SD = 208.46).

**Table 2:** **Correlations between measures of executive functioning and ERPs (*N* = 215)**.

	**1**	**2**	**3**	**4**	**5**	**6**	**7**	**8**	**9**	**10**	**11**	**12**	**13**	**14**
1. Stroop	–													
2. Go/nogo	0.03	–												
3. Compatibility reaction time	0.14^*^	0.05	–											
4. Letter-number sequencing	0.32^**^	−0.04	0.17^*^	–										
5. Backward digit span	0.27^**^	0.13	0.08	0.37^**^	–									
6. Sentence repetition	0.14^*^	0.02	0.07	0.37^**^	0.22^**^	–								
7. Wisconsin card sorting test	0.23^**^	0.05	0.13	0.38^**^	0.20^**^	0.17^*^	–							
8. Verbal fluency	0.40^**^	0.01	0.19^**^	0.40^**^	0.31^**^	0.30^**^	0.22^**^	–						
9. Letter monitoring	0.30^**^	−0.02	0.21^**^	0.42^**^	0.28^**^	0.14^*^	0.29^**^	0.26^**^	–					
10. P3b ERP mean amplitude composite	0.01	0.13	0.00	0.09	0.19^**^	0.06	0.11	0.08	0.13	–				
11. P3b ERP latency composite	0.13	0.02	0.08	0.01	0.05	0.10	−0.06	−0.03	−0.04	0.00	–			
12. N2 difference waveform mean amplitude composite	0.01	0.03	−0.02	−0.21^**^	−0.13^*^	−0.06	0.04	−0.03	−0.16^*^	−0.10	−0.05	–		
13. N2 difference waveform latency (incongruous—congruous)	0.05	0.00	0.04	0.17^*^	0.07	−0.05	0.10	0.00	0.11	−0.02	−0.12	−0.14^*^	–	
14. N2 difference waveform latency (reversed—congruous)	−0.21^**^	0.01	−0.10	−0.12	0.01	0.15^*^	−0.21^**^	−0.12	−0.08	−0.02	0.00	0.06	0.10	–

* p < 0.05;

** p < 0.01.

**Figure 1:**
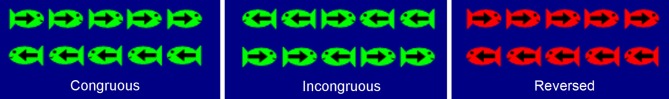
**The six flanker task stimuli used in the present experiment**.

In order to obtain two variables of each ERP component, participants also completed a modified visual flanker task (Rueda et al., 2004; Richardson et al., 2011) whilst EEG data were recorded. Each stimulus consisted of five fish presented on a blue background (see Figure 1). An arrow on the body of each fish indicated direction and the target was the central fish. Participants were instructed to press a response button situated on a keyboard (red felt patches on the “Z” and “/” keys) corresponding to the direction of the central fish. There were three conditions: in the congruent condition (0.5 probability), the five fish were green and all pointing in the same direction; an incongruent condition (0.25 probability), where all the fish were also green, however, the flankers pointed in the opposite direction to the central target; and a reversed condition (0.25 probability), in which the flanker fish were congruent, but all five fish were red, and required a response in the opposite direction to the central fish. Each fish subtended 0.9° horizontally and 0.6° vertically with 0.2° separating each fish and were randomly presented for 300 ms. A keyboard response was required before the next trial began. The task was presented as a game in which the participants had to feed the hungry central fish. Speed and accuracy were equally emphasized. A practice block of 8 trials was administered to ensure the participants understood the task requirements. A total of 352 trials were presented in two blocks.

### Electrophysiological acquisition

The EEG was continuously recorded using an Easy-CapTM. Electrodes were placed at 33 sites based on Easy-Cap montage 24 (excluding TP9 and TP10; see http://www.easycap.de/easycap/e/products/products.htm for more details). Eye movements were measured with bipolar leads placed above and below the left eye. The EEG was amplified with a NuAmps 40-channel amplifier, and digitized at a sampling rate of 250 Hz. Impedances were below 5 kΩ prior to recording. During recording, the ground lead was located at AFz and the right mastoid was set as reference. After recording, a linked mastoid reference was calculated offline, and Scan 4.3 was used to conduct the ERP processing. Offline, the EEG recording was digitally filtered with a 1–30 Hz zero phase shift band-pass filter (12 dB down). The vertical ocular electrodes enabled offline blink reduction according to the standard algorithm proposed by Semlitsch et al. (1986).

### ERP data analysis

Epochs encompassing an interval from 100 ms prior to the onset of the stimulus and extending to 1000 ms post-stimulus were extracted and baseline corrected around the pre-stimulus interval. Epochs containing artifacts larger than 150 μV or where an incorrect behavioral response was made were excluded from the ERP average. Additionally, the ERP data of participants who did not score significantly higher than chance on the congruous condition of the flanker task (*n* = 2) or had fewer than 25 acceptable epochs in any condition (*n* = 4) were excluded and treated as missing data. The average number of trials included in each grand-averaged waveform was 151 trials for the congruous condition, 71 for the incongruous condition, and 70 for the reversed condition.

**Figure 2:**
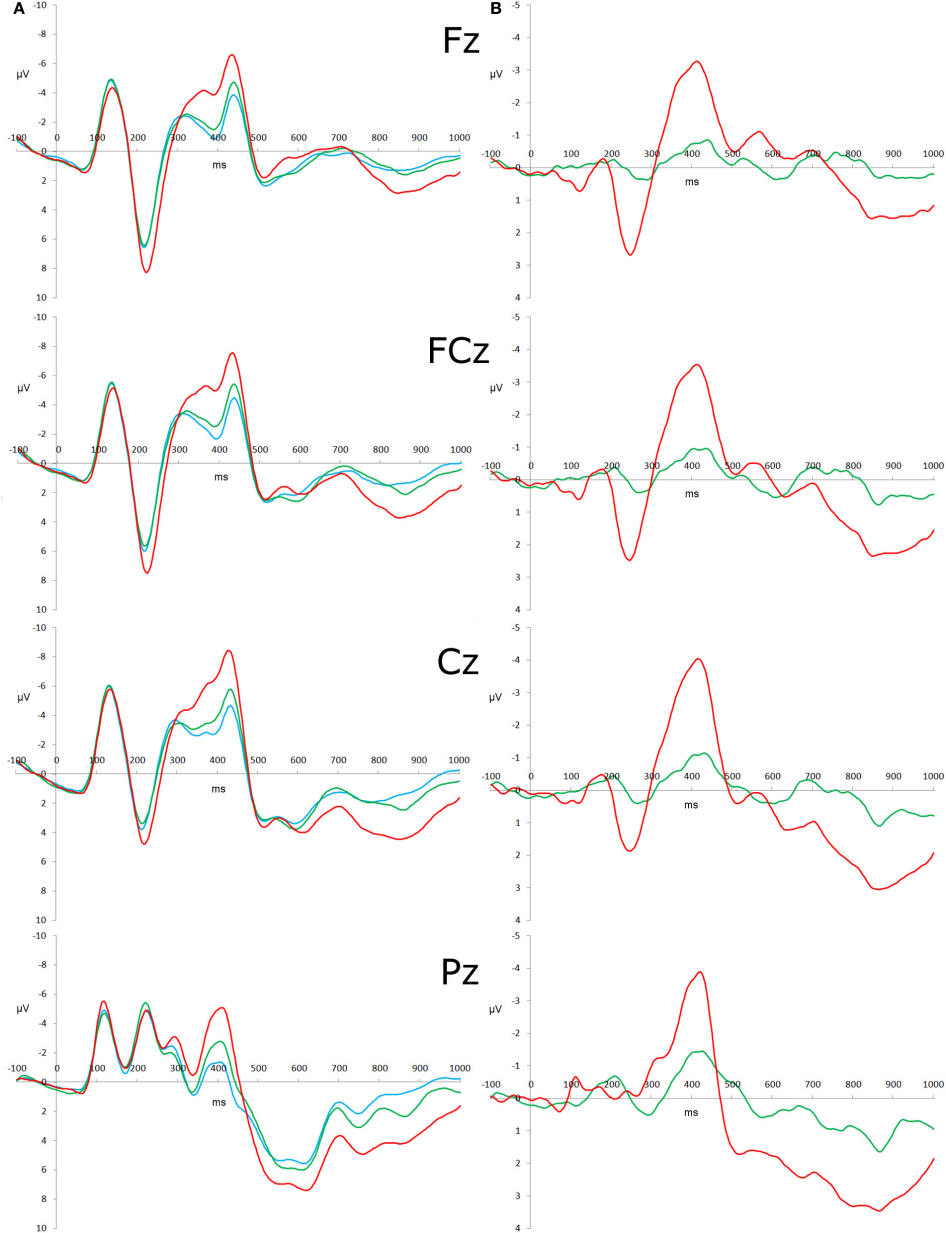
**Stimulus-locked ERP waveforms and difference waveforms. (A)** Grand-averaged ERP in response to congruous (blue), incongruous (green), and reversed (red) stimuli with the amplitude (μ V) as the y-axis and time (ms) as the x-axis. Time 0 represents stimulus onset. **(B)** Grand- averaged difference waveforms computed as the incongruous—congruous waveform (green) and reversed—congruous (red).

PCA with varimax rotation was used to determine the time windows of the P3b peaks. An epoch of 0–700 ms was used, with individual average waveforms from all three conditions at sites Fz, FCz, Cz, and Pz. The first extracted component was 584–648 ms (explaining 29.48% of the variance), matching a visual inspection of the P3b peaks (see Figure 2). Visual inspection of the individual participants' ERPs also revealed that not all of the participants displayed identifiable P3b peaks, so mean amplitudes were calculated across this interval. The N2 was calculated by extracting difference waveforms; that is, the individual ERP average from the congruous condition was subtracted from the individual ERP averages of the incongruous and reversed conditions. We calculated the interval over which the N2 inhibition effect was significant by comparing the amplitude of the difference waveforms at each time point from 0 to 600 ms against a mean value of zero. To control for the number of comparisons conducted, we required a successive sequence of 12 statistically significant values based on an autocorrelation of 0.9 and graphical threshold of 0.05, as detailed by Guthrie and Buchwald (1991). In the incongruous difference waveform, the N2 effect was significant over the interval 360–424, 348–468, and 348–472 ms at Fz, FCz, and Cz respectively. In the reversed difference waveform, the N2 effect occurred over the latency 316–496, 308–484, and 304–476 ms, at Fz, FCz, and Cz respectively. Visual inspection of the individual participants' ERPs also revealed that not all of the participants displayed identifiable N2 peaks in the difference waveforms, meaning that analyses on peak amplitude values were not possible. As a result, mean amplitudes were calculated across the interval 352–456 ms for the incongruous condition and 308–484 ms for the reversed condition, as these are the average latency windows for the two difference waveforms. Fractional area latencies for the P3b ERP components were measured by calculating the total positive area in the 584–648 ms measurement window (extracted by the PCA), and then determining the earliest latency at which the summed positive area exceeded 25% of the total (Hansen and Hillyard, 1984). The same process was used for the N2 difference waveforms, except examining the negative area in the two intervals mentioned above. Difference waveforms were calculated for the N2 components, but not for the P3b, because it is argued that the N2 is an index of response conflict. As there is no conflict in congruous condition of the flanker task, then it follows that any “extra” N2 amplitude is indicative of the response conflict presented within a trial (Van Veen and Carter, 2002; Nieuwenhuis et al., 2003, 2004; Lucci et al., 2013). Conversely, every trial of the flanker task requires the context to be updated, as new information is entering working memory (Polich, 2007). Hence, difference waveforms were not necessary.

### Transformation and outlier analysis

The transformation procedures for the eight executive function measures followed those conducted by Miyake et al. (2000), and are described in detail by Brydges et al. (2012b). Briefly, these were the use of arcsin transformations on proportion variables (Judd and McClelland, 1989), and a two-stage trimming procedure of scores that were more than 3 SDs from the mean in the compatibility reaction time task. Also, scores on all reaction time tasks, the Stroop task, and WCST were multiplied by −1 so that a higher score indicated better performance. When analyses were initially conducted, Heywood cases (i.e., models with standardized regression weights >1) occurred on each of the ERP latent variables (most likely due to multicollinearity, as correlations between indicators were generally very high). As a result of this, single indicator latent variables were created for each of the ERP factors, by adding the two related indicators together to form a composite variable (Landis et al., 2000) for each ERP index (i.e., the incongruous—congruous and reversed—congruous N2 amplitudes were added to make an N2 amplitude composite, the incongruous and reversed P3b amplitudes were added to make a P3b amplitude composite, and the incongruous and reversed P3b latencies were added to make a P3b latency composite). The N2 latencies were not included in the final analyses as every model with them included reported an inadmissible solution. Additionally, a single indicator latent variable could not be created with the two latencies as the correlation between them was very low (see Table 2). The other three composite variables all achieved a satisfactory level of normality without any transformations.

As CFA and SEM are very sensitive to outliers, univariate and multivariate outlier analyses were conducted on the eleven dependent variables. Specifically, a test score was considered a univariate outlier if it was greater than 3 SDs from the between-subjects variable mean, and was replaced with a value that was 3 SDs from the mean. This affected no more than 1.9% of the observations for each task. No multivariate outliers were identified when using a Cook's D value of >1 (Cook and Weisberg, 1982). Forty-eight participants had missing data for one or more tasks; however, Little's (1988) MCAR test was non-significant [χ^2^_(125)_ = 141.86; *p* = 0.14], indicating that the data were missing completely at random. These scores were estimated using the expectation maximization method.

### Statistical analysis

Amos 19 (Arbuckle, 2010) was used to estimate the latent variable models. In both CFA and SEM, several fit indices were used to evaluate the fit of each model to the data. The χ^2^ statistic is commonly used in latent variable analysis to measure goodness of fit; a non-significant χ^2^ indicates that data entered into a theorized model does not significantly deviate from the model, inferring good model fit (Blunch, 2008). Bentler's comparative fit index (CFI), the root-mean-square error of approximation (RMSEA), and the standardized root mean residual (SRMR) were also used to measure model fit. The criteria for excellent model fit based on these indices is greater than 0.95, less than 0.05, and less than 0.05 respectively (Blunch, 2008). Significance of correlation and path coefficients was determined using the same technique as Friedman et al. (2006). Specifically, χ^2^ difference tests were conducted when removing an individual parameter. If the difference was significant, it indicated that the removed coefficient was statistically significant, and should be kept in the model. This technique is more reliable than using test statistics that are based upon standard errors (Gonzalez and Griffin, 2001).

### Procedure

A maximum of 24 children at a time attended Project K.I.D.S. for two consecutive days over a two week period during the school holidays. All testing was presented in a child friendly manner, and each testing session lasted no longer than 25 min. Meals and activities (such as games and art) were scheduled between sessions to ensure the participants enjoyed themselves and did not become fatigued. All participants were given a Project K.I.D.S. t-shirt as a memento of their participation at the end of the second day.

## Results

### Descriptive statistics

Descriptive statistics of raw scores of the eleven measures (as well as Go/Nogo and flanker behavioral performance and N2 latencies) before any transformation procedures were conducted are presented in Table 1, and the correlations between the measures (after transformation, outlier analysis, and missing data estimation) are presented in Table 2. Additionally, the N2 amplitude variables were both found to be maximal at Cz, and the P3b component amplitudes were all maximal at Pz (see Figure 2).

### Latent variable analysis

To test that the P3b and N2 amplitudes and P3b latency are associated with a unitary executive function, a four-factor CFA was conducted with correlations between the P3b amplitude, N2 amplitude, P3b latency, and executive function factors allowed to vary freely and alternative nested models tested afterwards. The full four-factor model had very good model fit statistics (χ^2^ = 58.75, *df* = 44, *p* = 0.07, CFI = 0.94, RMSEA = 0.040, SRMR = 0.049). However, after testing the significance of parameter estimates, the best model only included correlations between P3b amplitude and executive functioning (*p* = 0.025), and N2 amplitude and executive functioning (*p* = 0.012). This final model had very good model fit statistics (χ^2^ = 61.94, *df* = 48, *p* = 0.09, CFI = 0.95, RMSEA = 0.036, SRMR = 0.051), and was not a significantly worse fit for the data than the full three-factor model (Δχ^2^ = 3.19, Δ *df* = 4, *p* = 0.53). All other correlations were non-significant (see Table 3).

**Table 3:** **Inter-factor correlations extracted from the CFA**.

	**1**	**2**	**3**	**4**
1. Executive function	–			
2. N2 Amplitude	−0.29^*^	–		
3. P3b Amplitude	0.19^*^	−0.19	–	
4. P3b Latency	0.00	−0.12	0.04	–

* p < 0.05.

**Figure 3:**
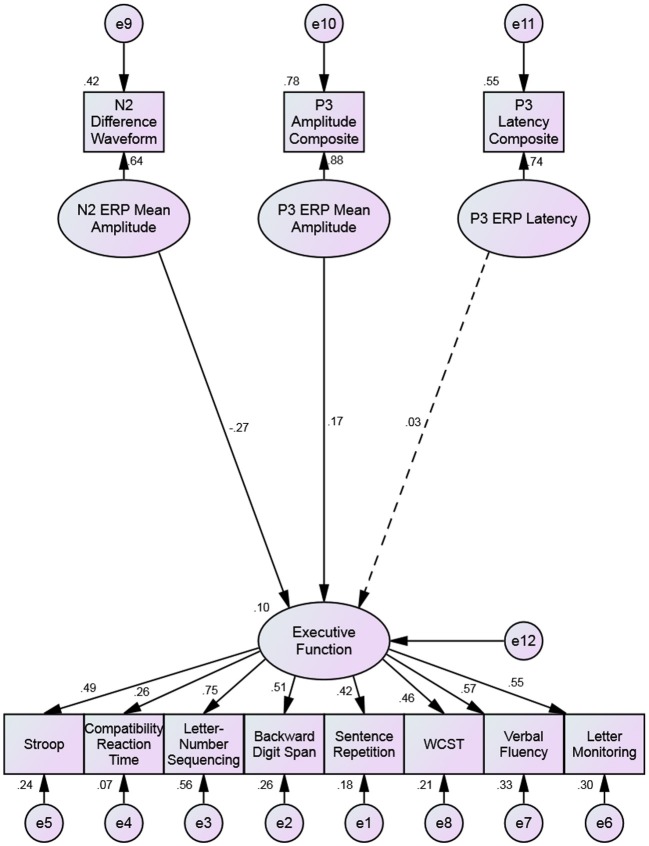
**Structural equation model predicting executive functioning with the N2 amplitude, P3b amplitude, and P3b latency**. Single-headed arrows have standardized factor loadings next to them. The dotted regression weight from the P3 latency factor to the executive function factor is non-significant. All other coefficients are significant to *p* < 0.05.

From this, an SEM was conducted, as this allows us to calculate the unique predictive contribution of each ERP factor on the executive function factor. The distinction between this analysis and the CFA is that SEM allows us to determine the unique contribution of each predicting factor after common variance has been accounted for. Figure 3 shows that, consistent with the findings of the CFA, both the P3b amplitude and N2 amplitude factors were significant predictors of the executive function factor, but the P3b latency factor was not (*p* = 0.049, *p* = 0.026, and *p* = 0.77, respectively).

## Discussion

The current study aimed to examine associations between components of the ERP and executive functioning. Previous research has reported associations between the P3b peak and working memory in both adults and children (Polich et al., 1990; Walhovd and Fjell, 2002), and between the amplitude of the N2 and response conflict/inhibition in adults and children (Jodo and Kayama, 1992; Van Veen and Carter, 2002; Cragg et al., 2009). The latent variable analyses used in the CURRENT study revealed that the N2 difference waveform and the P3b mean amplitudes are associated with executive functioning, but not the latency of the P3b component.

Previous research has found associations between behavioral performance on working memory tasks and P3b amplitude (Polich et al., 1990; Walhovd and Fjell, 2002), and between behavioral performance on response inhibition tasks and N2 amplitude (Jodo and Kayama, 1992; Cragg et al., 2009). The current study brings together this research with studies examining the structure of executive functions in children (Lehto et al., 2003; Brydges et al., 2012b). As individual executive functions (such as response inhibition and updating of working memory) are psychometrically indistinguishable in typically developing children in this age range (Hughes et al., 2009; Brydges et al., 2012b; Willoughby et al., 2012), it may follow that the ERPs associated with these specific executive functions are associated with a general executive function (the opposing view being that the respective ERPs will not be observable as the specific executive abilities are not sufficiently developed in this age group). The current study found that both the N2 difference waveform and the P3b amplitudes correlated with, and were predictive of, a unitary executive function in children. However, whilst the N2 amplitude associated with performance on Nogo tasks in adults is typically maximal at frontal scalp sites (Folstein and Van Petten, 2008), our results support the notion that the N2 amplitude is maximal at more central scalp sites in children (Jonkman, 2006).

It is also worth noting that the current analysis only used mean amplitude values of the two electrophysiological variables, as no clear peaks were identifiable. As a result of this, peak latency could not be included as a predictor of executive functioning, and it usually accounts for a significant proportion of variance in executive functioning (Walhovd and Fjell, 2002). However, when fractional areas latencies were calculated, no associations between latency and executive functioning were observed. This leads to the speculation that these ERPs begin to develop clear peaks around the same time as specific executive abilities develop, although, having said this, the deflections in the ERP were observable (even without any clear peak), yet the behavioral constructs were indistinguishable from each other. That is, whilst executive functioning is unitary in younger children, the N2 difference waveform and P3b component of the ERP are apparent. However, the change in the structure of executive functions, from unitary in children up to 9 years (Wiebe et al., 2008; Brydges et al., 2012b), to related yet separable functions in children aged around 11 years (Lehto et al., 2003; Duan et al., 2010) may be due to changes in the propagation of neural impulses—the peaks in the ERP become apparent before the specific behavioral abilities emerge. This could have important implications for the diagnosis of dysexecutive syndromes in samples where executive functions are psychometrically indistinguishable, as the ERP components may be a more sensitive measure of cognitive development. A longitudinal study would be required to test this conclusively.

Alternatively, using latent variables to test for associations between ERP components and executive functions in adults may be an informative area of future research. If the development of clear ERP peaks is associated with the development of abilities specific to single executive functions, then correlations between ERP and executive functioning factors should increase from the relatively low (but still significant; *r* = −0.29 and *r* = 0.19) values reported in this sample of children.

Additionally, the predictive power of other ERP peaks may have been missed in this study. For instance, Fjell et al. (2007) found associations between both P3a amplitude and latency (commonly associated with novelty detection, although generally not associated with any specific executive function) were both associated with higher-order cognitive functions in a sample of adults. Considering that the N2 difference waveform and P3b factors only accounted for 10% of the variance in the executive function factor (although both factors predicted a significant proportion of variance), it may be fruitful to also consider other predictor ERP peaks.

Another possible avenue of research involves examining differences between behavioral and electrophysiological development of individual executive functions. For instance, researchers have proposed taxonomies of both inhibition and working memory (Nigg, 2000; Oberauer et al., 2003). Specifically, Nigg proposed four subtypes of inhibition, which are all separate yet related constructs. From an electrophysiological perspective, previous research has suggested some common neural regions of activation, including the dorsolateral prefrontal cortex and the anterior cingulate cortex (Ridderinkhof et al., 2004; Chambers et al., 2006; Carter and Van Veen, 2007; Chambers et al., 2009). A few studies have examined two of these subprocesses (response inhibition and interference suppression), and have also found differential patterns of activation (Bunge et al., 2002; Brydges et al., 2012a, 2013). Bunge et al. reported multiple differences in regions of neural activation between task conditions requiring response inhibition and interference suppression. From a behavioral perspective, however, previous research has suggested that two of these subprocesses are actually indistinguishable (Friedman and Miyake, 2004). It may fruitful to further examine any potential differences (from both behavioral and neural perspectives) between subtypes of EFs such as inhibition to further our understanding of the architecture of EFs, and how these subtypes contribute to behaviors on complex tasks.

In conclusion, the present study has added evidence of the development of ERP correlates of executive functioning being observable before the specific executive functions themselves are psychometrically distinguishable. Additionally, evidence of the predictive qualities of ERPs on executive functioning from a latent variable perspective adds to the predominantly correlational-based knowledge of associations between brain and behavior (Polich et al., 1990; Walhovd and Fjell, 2002; Cragg et al., 2009). SEM analyses found that both the N2 difference waveform and P3b (thought to be electrophysiological correlates of response conflict/inhibition and updating of working memory, respectively) were significant predictors of executive functioning. Theories of developmental cognition would greatly benefit from the integration of neuroscientific techniques with behavioral evidence.

## Author contributions

Conceived and designed the experiment: Christopher R. Brydges, Mike Anderson and Allison M. Fox. Analyzed the data: Christopher R. Brydges and Allison M. Fox. Wrote the paper: Christopher R. Brydges. Reviewed final manuscript: Allison M. Fox, Corinne L. Reid and Mike Anderson. Trained and supervised ERP testers: Allison M. Fox. Conceived and designed the methodology for mass testing of children in a holiday program: Mike Anderson and Corinne L. Reid. Supervised the research group: Mike Anderson. Trained and supervised testers in child assessment and recruited participants: Corinne L. Reid.

### Conflict of interest statement

The authors declare that the research was conducted in the absence of any commercial or financial relationships that could be construed as a potential conflict of interest.
